# French good practice guidelines for medical and occupational surveillance of the low back pain risk among workers exposed to manual handling of loads

**DOI:** 10.1186/s40557-015-0069-9

**Published:** 2015-07-22

**Authors:** Audrey Petit, Jean-Baptiste Fassier, Sandrine Rousseau, Philippe Mairiaux, Yves Roquelaure

**Affiliations:** LUNAM University, Faculty of Medicine of Angers, Laboratory of Ergonomics and Epidemiology in Occupational health, Angers, France; Occupational Health Department, University Hospital of Angers, Angers, France; UMRESTTE, Claude Bernard University Lyon 1, Lyon, France; Intercompany Health Committee of Loiret, Loiret, France; Department of Occupational Medicine and Health Education, University of Liège, Liège, Belgium

**Keywords:** Guidelines, Low back pain, Manual handing of loads, Prevention, Surveillance

## Abstract

Several clinical practice guidelines related to the assessment and management of low back pain (LBP) have been published with varied scopes and methods. This paper summarises the first French occupational guidelines for management of work-related LBP (October 2013). There main originality is to treat all the three stages of primary, secondary and tertiary prevention of work-related LBP. The guidelines were written by a multidisciplinary working group of 24 experts, according to the Clinical Practice Guidelines method proposed by French National Health Authority, and reviewed by a multidisciplinary peer review committee of 50 experts. Recommendations were based on a large systematic review of the literature carried out from 1990 to 2012 and rated as strong (Level A), moderate (B), limited (C) or based on expert consensus (D) according to their level of evidence. It is recommended to deliver reassuring and consistent information concerning LBP prognosis (Level B); to perform a clinical examination looking for medical signs of severity related to LBP (Level A), encourage continuation or resumption of physical activity (Level A), identify any changes in working conditions and evaluate the occupational impact of LBP (Level D). In case of persistent/recurrent LBP, assess prognostic factors likely to influence progression to chronic LBP, prolonged disability and delayed return to work (Level A). In case of prolonged/repeated sick leave, evaluate the pain, functional disability and their impact and main risk factors for prolonged work disability (Level A), promote return to work measures and inter professional coordination (Level D). These good practice guidelines are primarily intended for professionals of occupational health but also for treating physicians and paramedical personnel participating in the management of LBP, workers and employers.

## Introduction

Manual handling of loads (MHL) is a widespread activity among workers: nearly a third of the European Union workers carry loads for at least a quarter of their working time [10]. MHL is ubiquitous and not specific to any particular business sector [29]. The most common injuries resulting from exposure to MHL are back injuries [5, 19, 23]. Although most workers recover completely after a back injury, about 2 to 7 % of them may develop chronic or recurrent low back pain (LBP). Repeated or prolonged sick leave for disease, occupational disease, work accident or disability due to LBP can compromise the worker’s subsequent employment prospects [3, 22, 38]. The problem of job retention for LBP workers is growing concern in the current socioeconomic context of industrialized countries. The changing work environment (more intense work, limited access to training, short-term jobs), ageing of the working population and longer careers are socio-demographic factors that require enhanced medical and occupational surveillance of workers exposed to MHL [31].

This manuscript summarizes the main recommendations for medical and occupational surveillance of the LBP risk among workers exposed to MHL of the French Society of Occupational Medicine (October 2013) [33]. These guidelines correspond to a constant concern of occupational risk prevention institutions of all industrialized countries of the world, as LBP at work is a major cause of invalidity. These guidelines are primarily intended for Occupational Physicians (OPs), specialized nurses and workplace risk prevention personnel. They are also intended for healthcare workers such as general practitioners (GPs) and spine specialists (rheumatologists, rehabilitation practitioners, orthopaedic surgeons, etc.), especially in terms of coordination of the management of workers with LBP and resolution of the obstacles to their return to work. Some guidelines may also concern paramedical personnel (physiotherapists, nurses, occupational therapists, psychologists). Finally, these guidelines are intended for workers, employers and stakeholders. The guidelines and the review are concerned with non-specific LBP unless stated otherwise. MHL is defined as “any transporting or supporting of a load, by one or more workers, including lifting, putting down, pushing, pulling, carrying or moving of a load, which, by reason of its characteristics or of unfavourable ergonomic conditions, involves a risk particularly of back injury to workers” by European legislation. The objectives of these guidelines are to define an appropriate surveillance strategy in order to detect and prevent low back injuries related to exposure to MHL. They apply to all countries’ workers exposed to MHL activities and especially concern the working-age adult population suffering from LBP and still at work, or suffering from LBP and on sick leave.

## Methods

### Literature review

The review methodology followed that of the French National Authority for Health clinical guidelines [13]. The present evidence review therefore started with a search for all published, methodologically sound, systematic reviews and international guidelines supplemented by narrative reviews and original scientific studies in key areas of interest or where systematic reviews were unavailable, in accordance with the French National Authority for Health methodology. The main target for the literature search was evidence from occupational settings or concerning occupational outcomes. The literature was searched systematically from January 1990 to March 2012 in several data bases: PubMed, Embase, NIOSHtic-2, Cochrane Library. The Key-Words was “(low back pain OR backache OR sciatica) AND (occupational health OR occupational medicine OR occupational disease OR occupational accident) AND (interventions OR prevention OR return to work OR absenteeism OR sick leave OR disability OR retirement OR employment OR job changes OR job adaptation OR job loss OR light duty OR ergonomic OR rehabilitation OR back school OR lumbar support)”. The final selection included five previous international guidelines, 159 systematic reviews, 34, meta analysis and 279 clinical trials. The main French-speaking ergonomics and occupational health journals were analysed for the period between 2005 and 2012. This research was completed by a review of websites, institutional reports, documentation from institutions in charge of occupational risk prevention and the documentation of French and European standards institutions and French and international standards bodies. More than 2800 titles and abstracts were considered. Detailed methodological information about search questions, the literature search, reviewing process and the consensus process are given in the guideline report [33].

### Scientific evidence and professional expertise

Guidelines were written by a working party and reviewed by a multidisciplinary peer review committee of 50 experts. The multidisciplinary working party comprised 24 experts and practitioners who had a good knowledge of professional practices in the field corresponding to the topic of the guidelines and were able to assess the relevance of published studies and the various clinical situations evaluated (occupational health physicians, rheumatologists, National health insurance consultant physicians, rehabilitation physicians, general practitioners, physiotherapists, ergonomists, occupational therapists, occupational nurses, regional health inspectors, chiropractors, occupational risk epidemiologists and work physiology and ergonomics scientists) (see acknowledgments, authors’ information and contribution). The working party met ten times from April 2012 to May 2013. Consensus was reached on all decisions regarding evidence reports and the specific recommendations. The independence and impartiality of the working party and review committee’s experts in relation to the topic of the guidelines were verified by a French National Authority for Health entity devoted to management of conflicts of interest.

On the basis of the data published in the literature and professional opinions, the proposed guidelines are classified as Level A, B, C or D according to the French National Authority for Health modalities (Table 1) and the Oxford grading system [28]. The absence of grading does not mean that the guidelines are not relevant and useful, but indicates the need to conduct further studies.

**Table 1:** Recommendation grading (according to the French National Health Authority, 2010 [28])

Level of scientific proof provided by the literature (for clinical studies)	Recommendation grading
Level 1	Level A
- High-power randomised comparative studies	Scientific proof established
- Meta-analysis of randomised comparative studies	
- Decision analysis based on well-conducted studies	
Level 2	Level B
- Low-power randomised comparative studies	Scientific proof presumed
- Well-conducted non-randomised comparative studies	
Level 3	Level C
- Case–control studies	Low level of proof
Level 4	Level D
- Comparative studies with major bias	Expert consensus
- Retrospective studies	
- Case series	

In the absence of studies, guidelines are based on a consensus between working party experts after consulting the peer review group

## Review

The resultant evidence is presented below under a logical sequence of occupational health situations about workers exposed to MHL: workers exposed to MHL without LBP, workers suffering from LBP and workers suffering from persistent or recurrent LBP.

### Individual information to be given to workers exposed to manual handling of loads

This information can be delivered by the OP or the occupational nurse, or other health professionals, depending on the occupation health and safety organisation and regulation. In every case, for workers exposed to MHL, the interaction with the health professionals can have direct positive effects, as erroneous beliefs may be identified and discussed [37]. It can also help to restore confidence to workers who are sometimes confused by contradictory information or medical advices [2, 5, 23, 34]. It is recommended to be particularly attentive to the content of the message delivered by the healthcare practitioner in view of its potential impact on the worker’s beliefs and behaviour (Level B) [2, 8, 34]; to emphasize the fact that LBP is common and frequently recurrent, but that episodes of LBP are usually brief with a spontaneously favourable outcome (Level B); to indicate that LBP has a multifactorial origin and that occupational factors are one of the modifiable factors influencing the incidence of LBP (Level B); to ensure the consistency of the risk prevention messages delivered by the occupational health team due to the negative impact of discordant messages (Level D).

For workers with LBP exposed to MHL, it is recommended to encourage continuation or resumption of physical activity and, when possible, work by taking into account the job characteristics and the possibilities of job adjustments (Level A) [5, 34, 39]; to backup oral information by written information complying with current recommendations (for example the “Back Book”) (Level A) [6, 14]; to provide information concerning physical demands of job (manual material handling, lifting, bending, twisting, and whole body vibration) and LBP, as it helps to improve the worker’s knowledge and promotes a positive change in their beliefs and their inappropriate behaviours (avoidance of movement) related to LBP (Level B) [37]; to explain and make less alarming any medical and technical terms in view of the absence of pathophysiological correlations in non-specific LBP, deliver reassuring information concerning prognosis, ensure that the worker has understood the essential messages and repeat the information concerning the general principles of occupational risk prevention (Level D).

### Medical surveillance of workers with LBP exposed to MHL

No scientific data are available to determine the optimal frequency of medical surveillance specific to the low back risk in symptomatic workers. For workers with LBP exposed to MHL, it is recommended that the frequency of follow-up be determined by the OP or the health professionals, according to the persistence of LBP, its physical and psychosocial impact on work, and job risk assessment (Level D).

Organic causes are rare among adults of working age. The first step in the evaluation of subjects with LBP, the so-called “diagnostic triage”, consists of confirming the non-specific nature of the LBP by eliminating any possible organic causes for LBP. The literature review identified a series of signs of medical severity (“red flags”), indicating a probability of an underlying organic cause for LBP that may justify complementary investigations [1, 5, 15, 18, 23–25, 34]. During the clinical interview of workers suffering from LBP, it is recommended to situate the current episode of LBP in the worker’s medical history (Level D); to look for an underlying specific cause of LBP, while keeping in mind that LBP secondary to a specific aetiology is rare (Level A) [15]; at the acute, subacute and chronic stages of LBP, look for medical signs of severity (“red flags”) allowing detection of an underlying disease (Level A) [1, 5, 15, 18, 23–25, 34]; to look for the presence of a radicular component associated with LBP (Level A) [34]. In the presence of a red flag and/or radicular pain, it is recommended to perform a specific clinical examination of the spine, regardless of the stage of the LBP (Level A) [1] and refer the worker to his/her GP for appropriate investigation and/or management (Level D).

In subjects aged 20 to 55 years with non-specific LBP, no laboratory tests or standard X-rays should be requested. However, in the presence of suspicious clinical signs (“red flags”), these complementary investigations (or even other second-line imaging examinations) are indicated and should be requested by the GP or spine specialist (Level A) [1, 5, 17, 26].

For LBP workers exposed to physical demands of work, occupational assessment is recommended to situate the current episode of LBP in the worker’s occupational history, and especially identify any changes in working conditions (Level D); to ensure that up-to-date job data are available (Level D); to evaluate the occupational impact of LBP (Level D); to assess, with the worker, the risks for his/her health, taking into account the job risk assessment, potential job adjustments and the medical and socioeconomic context (Level D). All in order to determine, in consultation with the worker, whether there is a need to recommend job adjustments and/or fitness for work restrictions; refer the worker to the general practitioner; adapt medical and occupational follow-up.

### Health surveillance in the case of persistent or recurrent LBP

When persistent or recurrent LBP is observed, it is recommended to evaluate prognostic factors, *i.e.* psychological and behavioural factors (“yellow flags”) likely to influence progression to chronic LBP and socio-economic and occupational factors (“blue and black flags”) likely to influence prolonged disability and delayed return to work. This evaluation can require several visits/interviews in complex cases (level B) [5, 7, 23, 26, 35]. Several occupational risk factors of prolonged work incapacity are also described in the literature [5, 7, 22, 30, 34, 35, 37]. It is recommended to evaluate the pain, functional disability and their impact (Level B) [34]. Evaluation of risk factors for chronic LBP or prolonged disability can help the clinician to learn more about worker capacities and the specific work situations and provide information to guide individual strategies to address them. The interview is recommended to situate the current episode of LBP in the employee’s medical and occupational history (Level D); to ask the employee to provide medical data concerning his/her LBP and its management (Level D); to evaluate the main risk factors for prolonged work disability (physical demands, quality of relationships and social climate, beliefs and behaviours related to pain, disability management policy) (Level D) [12, 35]; to assess the employee’s medical, administrative and socioeconomic situation (Level D) [35]; to ensure a shared understanding of the situation and the objectives of management between the employee, the family physician and the OP (Level D).

Several clinical screening tools can guide the examination and evaluation of risk factors of chronicity or prolonged disability (Table 2). To assess these factors, it is recommendations to use a visual analogue scale (VAS) to evaluate pain associated to LBP (Level A) [1, 34]. Risk factors for prolonged work disability can be evaluated by using the Örebro Musculoskeletal Screening Pain Questionnaire (OMPSQ) (Level D) [27]. The impact of functional disability related to LBP must be evaluated early and repeatedly (Level A) [34]. The main tools are the Roland-Morris, Oswestry and Dallas questionnaires (Level D), the Quebec scale and the SF-36 quality of life questionnaire (Level A) [1]. In the case of repeated or prolonged sick leave for more than 4 weeks, it is recommended to explicitly discuss with the worker his/her beliefs concerning the links between LBP and work (Level A). If a questionnaire approach is adopted, the Fear Avoidance Belief questionnaire (FABQ) questionnaire can be used, especially the FABQ-work subscale (Level D).

**Table 2:** Recommended tools for assessment of pain, functional and disability impact related to LBP

Assessed dimension	Recommended tool
Pain	Visual analogue scale (VAS) [34]
Work-related factors for prolonged disability	Örebro Musculoskeletal Screening Pain Questionnaire (OMPSQ) [16]
Functional disability	Roland-Morris questionnaire [32]
	Dallas Pain Questionnaire [20]
	Oswestry Disability Index [9]
	Quebec Back Pain Disability Scale [36]
	SF-36 quality life questionnaire [21]
Worker beliefs concerning the link between LBP and work	Fear Avoidance Belief Questionnaire (FABQ) work-subscale [11]

Clinical interview and physical examination can provide information on the biopsychosocial context of workers with chronic LBP, as they can reflect the subjective experience and impact of LBP. It is recommended to remind workers that they do not need to wait until a complete resolution of their symptoms before returning to work and that early return to work improves the prognosis, subject to job adjustments, when necessary (Level A) [2, 5, 23, 25]. During the occupational assessment, it is recommended to situate the current episode of LBP in the worker’s occupational history and look for any triggering or aggravating factors (Level D); to ensure that up-to-date job data are available (Level D); to estimate the worker’s capacity to return to work and conditions of return to work as a function of the previously evaluated occupational impact of LBP (Level D); to evaluate, together with the worker, the need to consider staying at work measures (Level D); to ensure a shared understanding of the situation and the objectives of management between the employee, the family physician and the OP (Level D). The worker must be at the centre of the staying at work approach. It is recommended to facilitate the worker’s transition from the health care setting to the workplace by encouraging and helping the worker to adopt a dynamic return to work, evaluating perceived physical demands and social support perceived by the worker and identifying the main difficulties related to work and possible job adjustments, in order to allow a transitional period for progressive and planned return to work and improve the worker’s capacity to cope with residual symptoms at work (Level C) [35].

Coordination of healthcare practitioners and social and occupational stakeholders has a positive influence on the return to work rate and on pain and disability of workers who return to work. This coordination can be facilitated by contact between healthcare workers and the OP, maintenance of a link between the workplace and the worker during the sick leave period, job analysis and possible proposal of job adjustments, consultation with occupational health professionals, and resolution of any medical, administrative or social problems [4, 35]. To improve coordination and return to work rate, it is recommended to evaluate, with the employee’s consent, the need for a consultation with the family physician, specialist(s) and, possibly, social insurance official and/or return to work coordinators (Level D); to ensure consistency of the messages delivered by the various personnel (Level D); to inform the employer, with the employee’s consent, about the desired conditions of return to work (Level D); to plan a workplace visit, in the presence of the worker whenever possible (Level D); to organize a meeting between the worker, supervisor, employer and, whenever possible, co-workers (Level D); to ensure application of the necessary measures to facilitate staying at work before the employee’s effective return to work (Level D).

The pre-return to work visit must be organised a sufficient time before the planned date of return to work in order to take any necessary measures before the worker return to work. Several pre-return to work visits may be necessary (Level D). Finally, *s*pecifically at the time of the return to work, it is recommended to assess, together with the worker, the risks for his/her health by taking into account the job risk assessment, any job adjustments required, staying at work actions taken and the medical and social context [4, 35] and determine the modalities of medical and occupational follow-up (Level D).

## Conclusions

Several clinical practice guidelines related to the assessment and management of LBP have been published in the past 10 years which varied in their scope and method. These ones are the first French occupational guideline for management of work-related LBP and their main originality is to treat all the three stages of primary, secondary and tertiary prevention of LBP for workers exposed to MHL. They are adapted to the French system of occupational health, which includes occupational health services employing occupational physicians and specialized nurses, but they are also intended for the surveillance of workers in other countries because they are also intended for treating physicians and paramedical personnel participating in the management of LBP. These recommendations are quite adapted to other occupational health legislations, especially for countries where medical surveillance of workers is insured by general practitioners (GPs). The literature synthesis recognizes some limitations because of the French National Authority for Health clinical guidelines method. It relies, as far as possible, on previous international clinical practice guidelines but also underlines insufficient evidence or limitations of current scientific investigations for several points of the topic.

These guidelines have been published by the French National Authority for Health clinical guidelines [33]. Their wide diffusion among the practitioners would improve the homogeneity of clinical practice in the management of LBP and promote a multidisciplinary approach of the three stages of LBP related to MLH’s prevention at the workplace.
